# Sequencing and molecular characterization of CTNCEC25, a China fixed rabies virus vaccine strain CTN-1 adapted to primary chicken embryo cells

**DOI:** 10.1186/1743-422X-11-176

**Published:** 2014-10-06

**Authors:** Shimao Zhu, Chunhua Wang, Pei Zhang, Hui Li, Shan Luo, Caiping Guo

**Affiliations:** Shenzhen Weiguang Biological Products Co., Ltd, Shenzhen, 518107 Guangdong province China

**Keywords:** Genome sequence, Rabies virus, CTNCEC25, Chicken embryo cell, Vaccine

## Abstract

**Background:**

Rabies virus is the main etiologic agent of the widespread neurological disease rabies. Recently, the China rabies virus vaccine strain CTN-1 adapted to chicken embryo cells, which has been designated as CTNCEC25, was obtained and demonstrated to have high immunogenicity. However, the full genome sequence of CTNCEC25 and its phylogenetic relationship with other rabies virus street and vaccine strains have not been characterized.

**Results:**

The complete genome of CTNCEC25 was sequenced and analyzed. The length of CTNCEC25 genome is 11,924 nucleotides (nt), comprising a 3′ leader sequence of 59 nt, nucleoprotein (N) gene of 1,425 nt, phosphoprotein (P) gene of 989 nt, matrix protein (M) gene of 803 nt, glycoprotein (G) gene of 2,067 nt, RNA-dependent RNA polymerase gene (L) of 6,474 nt and a 5′ trailer region of 71 nt. A comparison of the entire genomes of CTN-1 and CTNCEC25 identified 16 nt substitutions and 1 deletion, resulting in 8 amino acid (aa) changes in the five structural proteins with one in L (aa 1602), two in M (aa 99 and 191) and six in mature G (aa 147, 333, 389, 421 and 485). The percentage homology of the CTNCEC25 genomic sequence with other fully sequenced rabies virus strains ranged from 81.4% to 99.9%. Phylogenetic analysis indicated that CTNCEC25 was more closely related with those recently isolated China street strains than other vaccine strains. Virus growth analysis showed that CTNCEC25 achieved high rate of propagation in cultured cells.

**Conclusions:**

In this study, the complete genome of CTNCEC25 was sequenced and characterized. Our results showed that CTNCEC25 was more closely related to wild street strains circulating in China than other vaccine strains. Sequence analysis showed that the G protein ectodomain amino acid sequence identity between CTNCEC25 and other rabies virus strains was at least 90% identical. Furthermore, CTNCEC25 achieved high virus titers in cultured cells. Given that CTNCEC25 has high immunogenicity and induced strong protective immune response in animals, these results collectively demonstrated that CTNCEC25 is an ideal vaccine strain candidate for producing human vaccine with high quality and safety in China.

## Background

Rabies, which is an ancient global fatal central nervous system (CNS) disease, affects almost all kinds of mammals, including humans [1]. The mortality of rabies is almost 100%, and it is estimated that more than 55,000 people die from rabies worldwide annually, with about 95% of those deaths occurring in the developing world such as Asia and Africa (see WHO Fact Sheet No. 99). China has the second highest incidence of rabies after India, and a total number of 108,412 human rabies cases were recorded in China during the 55-year period between 1950 and 2004 [2].

*Rabies virus* (RABV) is the main causative agent of rabies and is the type species of the genus *Lyssavirus* of the family *Rhabdoviridae*. RABV has a non-segmented, single-stranded negative-sense RNA genome of approximately 12 kb that encodes five structural proteins in the order (3′ to 5′) of nucleoprotein (N), phosphoprotein (P), matrix protein (M), glycoprotein (G) and RNA-dependent RNA polymerase (large protein, L) [3, 4]. The viral RNA genome together with the N, P and L proteins forms a helical ribonucleoprotein (RNP) that is packaged into a bullet-shaped structure and wrapped by an envelope comprising an inner layer of the M protein and the transmembrane spike G protein [1]. While the RNP complex is the entity responsible for viral transcription and replication within the cytoplasm of the host cell, the G and M proteins play pivotal roles in viral assembly and budding [5, 6].

The RABV G protein is the only viral protein exposed on the surface of the virus. Previous studies have established that G protein is not only the major determinant of viral pathogenicity but is also the major protective antigen that induces the production of virus-neutralizing antibodies (VNAs) responsible for the immune responses of the host [7–10]. Moreover, the G protein is also involved in the neurotropism of RABV [11–18]. A number of antigenic sites to which neutralizing monoclonal antibodies bind were mapped in the G protein, including antigenic site I (aa 231), II (aa 34 - 200), III (aa 330 - 357), IV (aa 264) and “a” (aa 342 - 343) [19]. In addition, a linear epitope named G5 was also identified in the G protein (aa 244 - 281) [20, 21]. Among these antigenic sites, aa 147 and 333 have been shown to be critical for G protein function as mutation in either of these two sites significantly affected RABV antigenicity and pathogenicity [22, 23]. Furthermore, a region between aa 164 to 303 of the Nishigahara strain G protein also plays an important role in virus pathogenicity for adult mice, with aa 242, 255 and 268 constituting the key residues [24, 25].

Currently, the pathogenesis of RABV has not been fully elucidated and vaccination is the only effective method to protect against RABV infection. Since the first development of a rabies vaccine by Pasteur in the late 19th century, vaccination has been widely used in both domestic animals as well as reservoir species [26, 27]. At present, a number of RABV strains were used for vaccine production in different countries. Four virus strains, CTN-1, aG, PM and PV, have been applied in human rabies vaccine production in China and CTN-1 and aG strains are Chinese domestic isolates [28]. The CTN-1 strain was first isolated from brain tissue of a patient with rabies from Zibo, Shandong province while the aG strain was obtained from a rabid dog in Beijing [28]. However, although both the CTN-1 and aG strains are indigenous to China, they have distinct phylogenetic relationship. Previous studies suggested that the aG strain was more closely related with strains in northern and northeast part of China and phylogenetic analysis based on the N gene showed that the aG strain mainly clustered with strains from Japan and America but distantly clustered with most China street strains while the CTN-1 strain clustered preferentially with China native street viruses [29, 30], suggesting that the CTN-1 strain had closer genetic relationship with street viruses prevailing in China. It has been assumed that the efficiency of cross protection against the epidemic street virus conferred by rabies vaccine correlated with the homology between the vaccine strain and the challenge strains [31]. Therefore, the CTN-1 strain is theoretically more suitable for vaccine production than the aG strain in China.

Recently, a CTN-1 strain adapted to chicken embryo cells (CECs), which has been named CTNCEC25, was successfully obtained and demonstrated to have high immunogenicity and potency to induce a strong protective immune response in animals [32]. Besides, CTNCEC25 lost pathogenicity to adult mice by intracerebral inoculation [32]. In the present study, to gain more insight into the biological characteristics of CTNCEC25, the complete sequence of the CTNCEC25 strain was sequenced and characterized. Sequence comparison and phylogenetic analysis demonstrated that CTNCEC25 was more closely related with those recently isolated China RABV street strains than other vaccine strains commonly used in China. Virus growth curve showed that CTNCEC25 replicated stably and maintained high titers at cultured cells. Therefore, these results demonstrated the potential use of the CTNCEC25 strain for producing human rabies vaccine in China.

## Results

### The genome organization of the CTNCEC25 strain

Based on the nucleotide sequence determined using a total of 13 primer pairs (as shown in Table 1), the complete sequence of the CTNCEC25 strain was obtained and submitted to the NCBI GenBank (GenBank accession no. KJ466147). The genome length of CTNCEC25 was 11,924 nucleotides (nt) and the overall organization of the CTNCEC25 strain was similar to that of the parental CTN-1 strain except that it has one deletion in the poly A tail of the P gene and is summarized as follows: a 3′ leader region of 59 nt (1 - 59), N gene (60 - 1,484), P gene (1,487 - 2,475), M gene (2,481 - 3,283), G gene (3,289 - 5,355), L gene (5,380 - 11,853), and the 5′ trailer region of 71 nt (11854 - 11924). The coding sequence (CDS) of the five structural proteins are located as follows: 1,353-nt N protein (72 - 1,424), 894-nt P protein (1,516 - 2,409), 609-nt M protein (2,496 - 3,104), 1,575-nt G protein (3,316 - 4,890) and 6,387-nt L protein (5,407 - 11,793).

**Table 1:** **Primers used in this study**

Primer	Sequence (5′ to 3′)	Base position (bp) ^***a***^
LH-1 F	TACGCTTAACAACCAAATCAAAGA	1-1075
LH-1 R	GGTGCACATGCGGCAATA	
LH-2 F	TCCGTTCATTAGGCCTGAGTG	937-2038
LH-2 R	CTGCCATTCGGGCTTTTG	
LH-3 F	GGAAGATCCTCGGAGGACAA	1927-2979
LH-3 R	TTGTCTTGCACTCACTCTTATCTG	
LH-4 F	GAGGGCATGAACTGGGTGTA	2818-3890
LH-4 R	GGTTGGTAGAGCAGTAGGCAGAA	
LH-5 F	TCATTGGCTCCGGACTGTAA	3730-4766
LH-5 R	TAGTCCTTCGGCAACACGTC	
LH-6 F	CTCAGGGGTTGATCTCGGTCT	4654-5735
LH-6 R	CAGAGTGAGCACCATACAACCA	
LH-7 F	AGACCTTACCGGATGACTTTGAC	5585-6623
LH-7 R	TCGCCAAGCACTCCTGATA	
LH-8 F	GTTATAGACATTGGGGGCATCC	6510-7599
LH-8 R	TTGTCTCCTTGCGCCAGTAT	
LH-9 F	TGGAAGGCTTACGGCAGAA	7494-8661
LH-9 R	GGTGTCTGAGTCATCCGGTTG	
LH-10 F	TCGGACGATCAGAAGGCAGTT	8560-9601
LH-10 R	TGAGTCATGTATCGCGACCAAG	
LH-11 F	CTGATATCCGCCTGAAGCCTG	9486-10630
LH-11 R	ATGTGTTCCGGATGCCATCA	
LH-12 F	GGAGGAATATCGCGAGCAGTA	10532-11686
LH-12 R	AATACTCCCGACAAGTCTGGTG	
LH-13 F	GTTGGTCTGACGATACCTCAGTGT	11565-11925
LH-13 R	TACGCTTAACAAAAAGACCATAAAGATGA	

^*a*^Position from the 5′ terminal region of the full-genome sequence of the CTN-1 strain.

The comparison of the complete CTNCEC25 genomic sequence with those selected RABV strains available in the GenBank was performed to investigate the relative similarity of the CTNCEC25 strain to other RABV strains (Table 2). The results showed that the percentage homology of the CTNCEC25 strain at nucleotide level with other full-length genomes ranged from 81.5% with SHBRV-18 strain to 99.9% with the parental CTN-1 strain, indicating high relatedness of the CTNCEC25 strain with other RABV strains (Table 3).

**Table 2:** **The RABV strains for which complete genome or the G gene sequence is available that were used in this study**

	Virus strain	Accession no.	Isolate country	Length (bp)
Vaccine strains	CTNCEC25	KJ466147	China	11,924
	CTN-1	FJ959397	Zibo, China	11,925
	SRV9	AF499686	China	11,928
	SAG2	EF206719	France	11,928
	ERA	EF206707	Germany	11,931
	SAD B19	M31046	America	11,928
	HEP-Flury	AB085828	America	11,615
	FluryLEP	DQ099524	Germany	11,711
	PM1503	DQ099525	Germany	11,723
	PV	M13215	France	11,932
	RV-97	EF542830	Russia	11,932
	3aG G	L04522	Beijing, China	1,575
Street strains	HN10	EU643590	Hunan, China	11,923
	BD06	EU549783	Hunan, China	11,924
	DRV	DQ875051	Jilin, China	11,863
	MRV	DQ875050	Henan, China	11,869
	FJ008	FJ866835	Fujian, China	11,924
	D01	FJ712193	Zhejiang, China	11,925
	JX08-45	GU647092	China	11,922
	DRV-AH08	HQ450385	Anhui, China	11,924
	SHBRV 18	AY705373	America	11,923
	8743THA	EU293121	Thailand	11,923
	NNV-RAB-H	EF437215	India	11,928
	Nishigahara	AB044824	Japan	11,926
	HM65	AY257980	Thailand	1,575
	CHAND03	AY987478	India	1,592
	MAL1-HM	AF325487	Malaysia	2,091
	BeijingHu1 G	EU700029	Beijing, China	1,575
	GuizhouA10(H) G	EU267745	Guizhou, China	1,575
	Hebei0(H) G	EU267752	Hebei, China	1,575
	HNDB28 G	EU008927	Hunan, China	1,575
	Hubei0703081 G	EF643518	Hubei, China	2,064
	WG430 G	DQ849060	Hunan, China	1,575
	WG432 G	DQ849059	Hunan, China	1,575
	Yunnan_MD06 G	EU253477	Yunnan, China	1,575
	Zhejiang Wz1(H) G	EF556198	Zhejiang, China	2,067
	FY1 G	DQ849044	Anhui, China	1,575
	Yue1 G	DQ849070	Guangxi, China	1,575
	Jiangsu Wx0(H) G	EU267772	Jiangsu, China	1,575
	NC G	DQ849064	Jiangxi, China	1,575
	CQ92 G	DQ849072	Chongqing, China	1,575
	Yunnan_Qj07 G	EU275240	Yunnan, China	1,575
	YunnanTc06 G	EU275242	Yunnan, China	1,575

**Table 3:** **Comparison of genome sequence similarity between CTNCEC25 and several RABV strains**

Strains	Similarity (%)	Strains	Similarity (%)
CTN-1	99.9	HN10	93.4
SRV9	84.4	BD06	88.1
SAG2	84.4	DRV	83.7
ERA	84.6	DRV-AH08	88.0
SAD B19	84.4	MRV	84.2
HEP-Flury	84.8	SHBRV18	81.5
FluryLEP	84.9	FJ008	88.2
PM1503	84.5	D01	88.2
PV	84.4	JX08-45	93.4
RV-97	83.8	8743THA	88.4
Nishigahara	83.7	NNV-RAB-H	84.8

### Sequence comparison of the genome of CTNCEC25 and CTN-1

To investigate in detail the sequence variation after the adaption of CTN-1 to CECs, the genome sequence of CTNCEC25 and the parental CTN-1 strain was compared and analyzed (Table 4). Compared to that of the CTN-1 strain, a total of 16 nucleotide substitutions and 1 deletion, which resulted in 8 aa residues changes in the five structural proteins, were identified in the CTNCEC25 strain. The G gene was the most varied gene of the CTNCEC25 strain with 7 nucleotide mutations. All the seven mutations except one in the G gene non-coding region (5251 nt), were non-synonymous which changed 5 aa residues in the G protein. In contrast, although the L gene possessed 6 nucleotide mutations, only one of them affected the amino acid sequence. In addition, the N gene has 1 synonymous mutation and the P gene has a deletion of one A nucleotide in the potential polyadenylation signal. The M gene has 2 mutations which affected 2 aa residues in M protein.

**Table 4:** **Comparison of the sequence difference between the genomes of the CTNCEC25 and CTN-1 strains**

Genome location ^***α***^	Protein position	Nucleotide/amino acid substitution (CTN-1 → CTNCEC25)	
461	N	G → A/-^*b*^	
2476	P	G → -^*c*^/-	
2792	M	T → G/Leu → Arg	
3068	M	C → T/Ser → Leu	
3812	G-147	A → G/Lys → Glu	
4371	G-333	G → A/Arg → Gln	
4538	G	G → A/Glu → Lys	
4635	G	C → A	/Pro → Gln
4636		A → G	
4826	G	T → C/Ser → Pro	
5251	G	C → A/-	
6289	L	A → G/-	
7078	L	G → A/-	
7750	L	G → A/-	
9886	L	G → T/-	
10141	L	A → G/-	
10212	L	G → A/Arg → Lys	

^*α*^Position from the 5′ terminal region of the full-genome sequence of the CTN-1 strain.

^*b*^Amino acid synonymous mutation.

^*c*^Nucleotide deletion.

**Figure 1:**
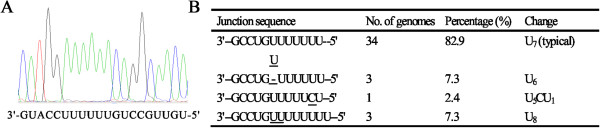
**Analysis of sequence across the gene junctions of P and M genes. (A)** Sequence analysis of the PCR product of the P and M gene junction in CTNCEC25. **(B)** Intergenic junction sequences of the P and M genes in 41 RABV genomes.

The finding that a deletion of one A nucleotide was occurred in the gene junction of P and M was unexpected as several studies have shown that a 3’- U-U-U-U-U-U-U-5’ (U7) tract at the end of each gene was essential for the polyadenylated tail of the five structural protein genes and was well conserved in all these five structural protein genes [3, 33]. To further investigate the heterogeneity of the gene junctions of P and M, the CTNCEC25 P gene was used to searched against the nucleotide sequences at the NCBI database and a total of 41 RABV strains with complete genome sequenced was selected to compare the sequence heterogeneity of the P-M gene junctions. The summary of the sequence analysis is shown in Figure 1. The sequence across the P-M junction was wild type (with U_7_ tract) in 34 of 41 genomes and the shortening of U_7_ tract to U_6_ was found in three genomes including CTNCEC25. In the rest four genomes that were not wild type, the U tract was extended to U_8_ in three and interrupted by a C residue (U_5_CU_1_) in one genome (Figure 1). The above data showed that mutations that are known to eliminate termination of transcription might be selected and preserved in virus population during evolution.

### Structural features of the G protein of the CTNCEC25 strain

As the G protein is essential for viral pathogenicity, the G protein amino acid sequence variation between CTNCEC25 and CTN-1 was investigated. Compared to the CTN-1 G protein, amino acids Lys_147_, Arg_333_, Glu_389_, Pro_421_ and Ser_485_ were changed to Glu_147_, Gln_333_, Lys_389_, Gln_421_ and Pro_485_, respectively, in the CTNCEC25 G protein (Table 4). Among these amino acids, Lys_147_ and Arg_333_ have been shown to be essential for the pathogenesis of RABV [11, 34–38]. The results also imply that antigenicity would be affected in CTNCEC25.

To investigate the G protein ectodomain sequence identity of CTNCEC25 with other RABV street or vaccine strains, pairwise comparisons using the G protein ectodomain amino acid sequence was performed. The results showed that the identity of the G protein ectodomain sequence ranged from 90.0% to 99.1% between CTNCEC25 with other RABV strains (Table 5).

**Table 5:** **Homologies of the ectodomain amino acid sequences of the CTNCEC25 G protein with those of other virus strains**

Strain	Identity (%)	Strain	Identity (%)
CTN-1	99.1	SHBRV 18	90.9
SRV9	93.8	BeijingHu1	95.9
SAG2	94.5	GuizhouA10(H)	94.1
ERA	94.8	Hebei0(H)	94.8
SAD B19	94.5	HNDB28	95.9
HEP-Flury	95.2	Hubei0703081	95.7
FluryLEP	94.8	WG430	95.7
PM1503	93.8	WG432	95.7
PV	94.8	Yunan_MD06	95.2
3aG	93.6	Zhejiang Wz1(H)	95.7
HN10	97.9	FY1	95.4
BD06	95.2	Yue1	95.4
DRV	90.0	Jiangsu Wx0(H)	94.5
MRV	93.2	NC	95.9
FJ008	95.4	CQ92	97.0
D01	95.4	Yunan_Qj07	95.2
JX08-45	96.6	YunnanTc06	95.7
RV-97	92.5	MAL1-HM	96.1
Nishigahara	94.1	CHAND03	94.5
8743THA	95.2	NNV-RAB-H	96.1

### Phylogenetic analysis of the CTNCEC25 strain with other RABV street and vaccine strains

**Figure 2:**
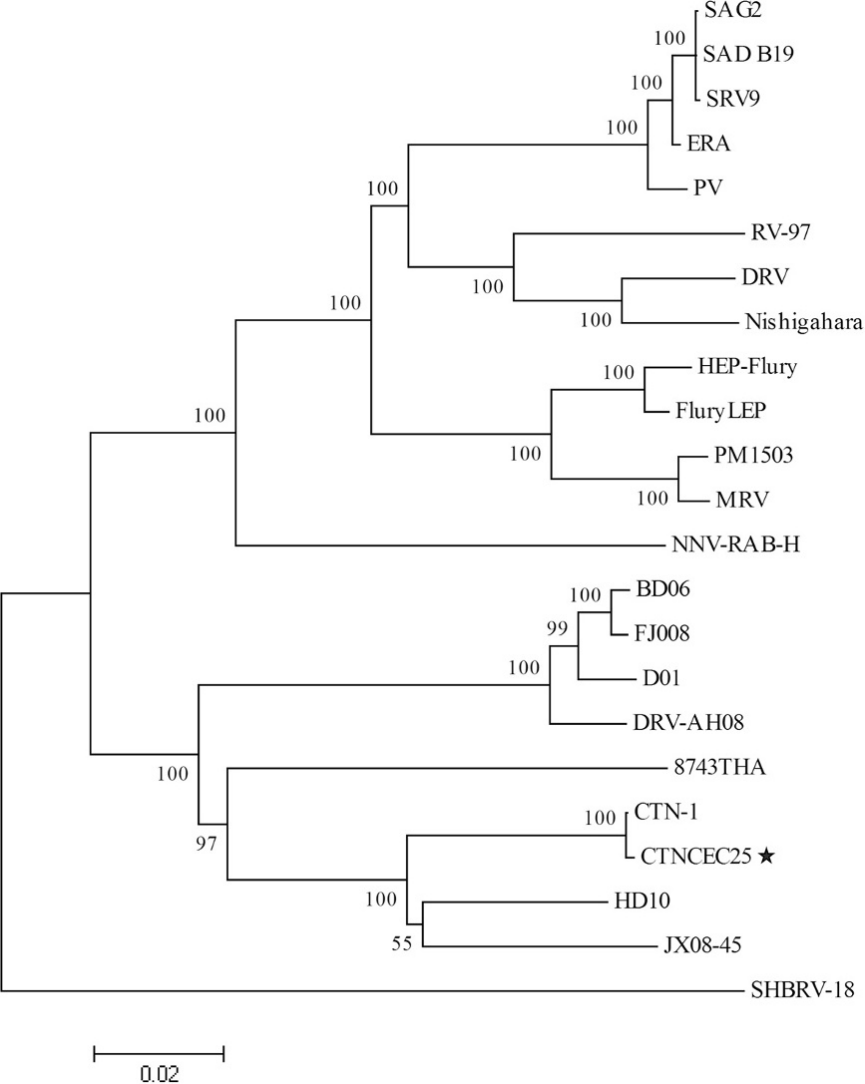
**Phylogenetic analysis of the complete genome sequence of CTNCEC25 compared with other RABV strains.** The tree was constructed using the Neighbor-Joining algorithm in MEGA 4.0 software. The numbers below the branches are bootstrap values for 1000 replicates. CTNCEC25 was marked with a black star.

**Figure 3:**
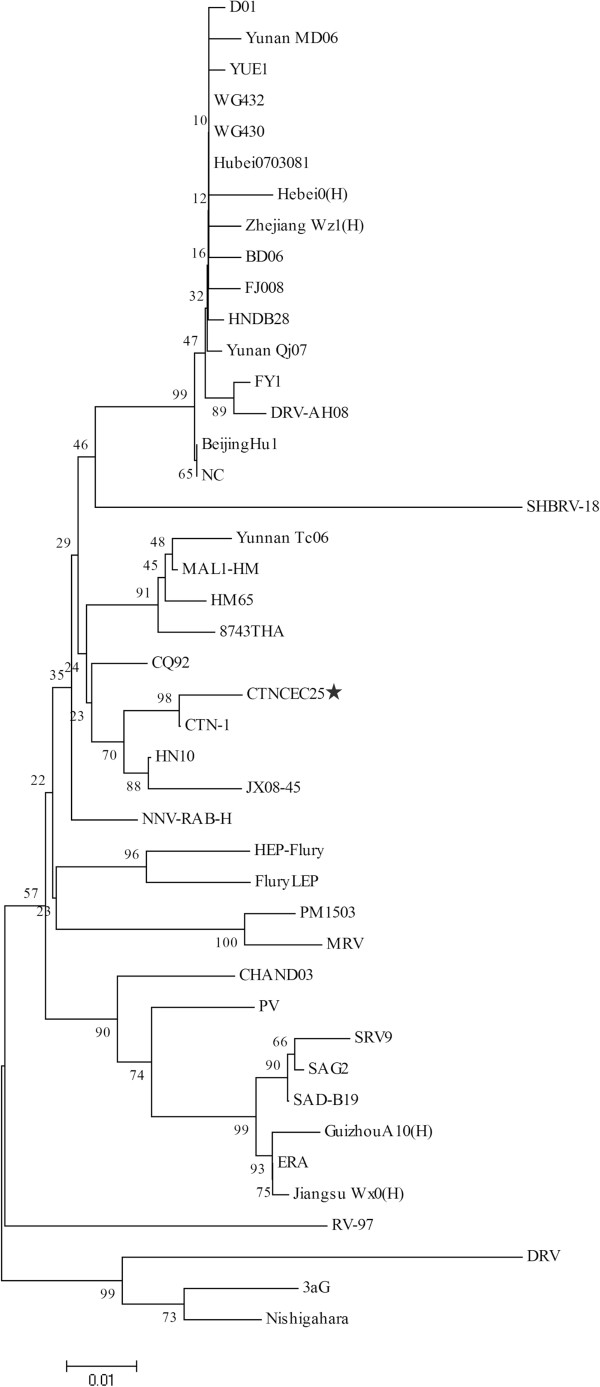
**Phylogenetic analysis of the mature G protein amino acid sequences of CTNCEC25 compared with other RABV strains.** The tree was constructed using the Neighbor-Joining algorithm in MEGA 4.0 software. The numbers below the branches are bootstrap values for 1000 replicates. CTNCEC25 was marked with a black star.

To further determine the phylogenetic relationship of the CTNCEC25 strain with others RABV strains, especially those recently isolated from different regions in China, two phylogenetic analyses were performed using either the complete genome sequences (Figure 2) or the mature G protein amino acid sequences (Figure 3) of the CTNCEC25 strain and others RABV strains, mainly those recently isolated street strains circulating in China.

As expected, at the genomic level, the CTNCEC25 strain was most closely related with the parental CTN-1 strain (Figure 2 and 3), which was consistent with genome comparison analysis (Table 3). Furthermore, compared to other vaccine strains used in China, such as PM and PV, the CTNCEC25 strain was more closely related with those RABV street strains. This phenomenon was even more pronounced when the phylogenetic tree was built based on the mature G protein amino acid sequences, in which the CTNCEC25 strain was clustered together with almost all of the selected recently isolated China RABV street strains while other commonly used vaccine strains were clustered into another group together with only a few China RABV street strains (Figure 3). Therefore, the above results suggested that the CTNCEC25 strain was phylogenetically more closely related with those native China RABV street isolates than other vaccine strains commonly used in China.

### Virus replication in cell culture

**Figure 4:**
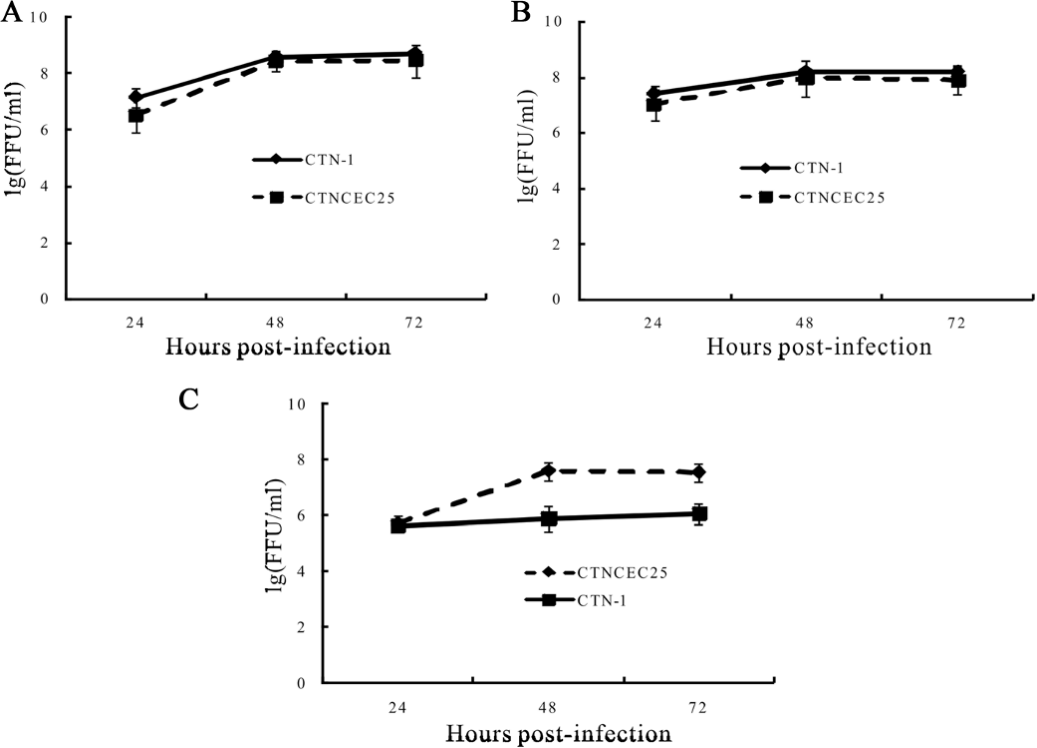
**Growth curve analysis of the CTNCEC25 and CTN-1 strains in Vero (A), NA (B) or CECs (C).** Cells were infected with indicated virus at an MOI of 3 FFUs/cell. At different times p.i., the virus in the supernatant was harvested and titrated in BSR cells. Each data point was determined from the average for three independent infections. Error bars represent standard deviations.

To compare the infectivities of the CTN-1 and CTNCEC25 strains in both neuronal NA cells and the nonneuronal Vero or CECs, viral replication was examined by analysis of progeny virus production in NA, Vero or CECs infected with CTN-1 or CTNCEC25 at a multiplicity of infection (MOI) of 3 fluorescent focus units (FFUs)/cell. As illustrated in Figure 4A and B, the viral titers of the CTNCEC25 strain in both NA and Vero cells were comparable to that of the parental CTN-1 strain and similar growth kinetics were observed for these two viruses with peak titers at 72 hours post infection (h p.i.) reaching about 10^8.0^ FFUs/ml. As expected, CTNCEC25 replicated at a similar robustness and reached high titers in CECs, although the titers were slightly lower than that in NA or Vero cells (Figure 4C). On the other hand, as the CTN-1 strain was previously maintained in Vero cells, it replicated poorly in CECs and the titers at 72 and 96 h p.i. were approximately 100-fold lower than that of the CEC-adapted CTNCEC25 strain (Figure 4C). Therefore, the results indicated that the adaptation of CTNCEC25 to CECs did not affect virus replication in cultured cells.

## Discussion

In the present study, the complete genome of the RABV strain CTNCEC25, the first CTN-1 strain adapted to CECs, was sequenced and analyzed. The results demonstrated that the CTNCEC25 strain was closely related to China RABV street strains recently isolated from different regions. Furthermore, although the CTNCEC25 strain achieved stable and high titers in cultured cells and CECs (Figure 4), it caused no lethality in adult mice by intracerebral inoculation [32], thus providing a rationale for its potential use for human vaccine production in China.

Comparison of the nucleotide sequences of CTNCEC25 with CTN-1 identified that all nucleotide changes occurred in the structural protein genes, with the G gene being the most variable. Similar results were observed in another attenuated RABV strain, RC-HL, which was derived from the RABV Nishigahara strain after 330 passages in chicken embryos and cell cultures [24]. It has been shown that the G gene was the most variable when comparing the complete genome sequences of the RC-HL strain and the Nishigahara strain [39]. Given that RABV is highly neurotropic in nature and the fact the G protein is the major structural protein involved in the neurotropism of RABV by recognizing receptors on neurons, it is therefore not unexpected that the G protein underwent greater selection pressure during adaptation to cultured nonneuronal cells.

Previous studies have identified several amino acids in G protein that were important for the antigenicity and pathogenicity of RABV [22, 23]. In the present study, two of these critical amino acids, aa 147 and 333, were found to be mutated in CTNCEC25 G protein during adaptation to CECs. Therefore, it was assumed that the pathogenicity of CTNCEC25 may be severely attenuated in adult mice, which was consistent with our previous *in vivo* study showing that CTNCEC25 was apathogenic to adult mice by intracerebral inoculation [32].

Sequence analysis identified that the *Lyssavirus* genome contains the signals essential for the transcription initiation, termination and processing for all the five structural protein genes, and the RABV is no exception [4]. A consensus sequence, 3’-A/U-C-U-U-U-U-U-U-U-5’, is conserved in all of the five RABV structural protein genes [3]. Several studies using *Vesicular stomatitis virus* (VSV), the prototype of the *Vesiculovirus* genus, showed that the U_7_ tract is strictly conserved and essential for VSV mRNA termination and polyadenylation, and either shortening or interrupting it with a heterologous nucleotide eliminates mRNA termination and polyadenylation [40, 41]. As is the case for CTNCEC25, however, the U_7_ tract is only conserved in four of the five structural protein genes, N, M, G and L, but not the P gene, in which the U_7_ tract was shortened to U_6_. Therefore, it is assumed that the expression of M gene, which is located downstream of the P gene, would be affected in CTNCEC25 due to the read-through of the upper P gene. Previous studies have revealed that the M gene encodes a multifunctional protein that plays essential roles not only in mediating viral assembly and budding but also in regulating the balance between the transcription and replication of RABV. So the disruption of M gene expression should certainly impair the CTNCEC25 replication in cultured cells. Although we did not perform transcriptional analysis of the CTNCEC25 M gene, this possibility could be ruled out as the growth kinetics of CTNCEC25 in cultured cells were indistinguishable from that of CTN-1 (Figure 4).

After careful inspection of the database, we found that while the typical U_7_ tract was the preponderant sequence at the P-M junction, several types of disruption of the typical U_7_ tract were observed, although with a low frequency, in the P-M junctions, including shortening or lengthening of U_7_ tract to U_6_ or U_8_ and interruption of the U_7_ tract by a different nucleotide (Figure 1). Therefore, it is possible that the RABV street strains have accumulated mutations during evolution and maintained these mutations to increase their population diversity, better adapt to their hosts or disseminate infection to a new host species. On the other hand, it also cannot rule out the possibility that different mechanisms may exist upon the molecular biology between RABV and VSV, as RABV and VSV share distinct natural histories and pathogenicity despite the close relationship within each other [4]. Further studies are needed to unravel the mechanisms underlining the regulation of gene expression of CTNCEC25.

Phylogenetic analysis using the genome sequence or the mature G protein amino acid sequence identified that CTNCEC25 shared high homology with wild strains isolated from different regions in China. It has been previously reported that the identity of the ectodomain amino acid sequence of RABV G protein directly correlated with the efficacy of vaccination and VNAs displayed cross-protection only when the amino acid sequence of the G protein ectodomain was at least 74% identical [31]. The recent antigenic analysis using serological assay data has also demonstrated that a 4.8% change in the G protein ectodomain amino acid sequence would cause a change of one antigenic unit between viruses (equivalent to a two-fold change in antibody titer) and there is a generally good correlation between genetic distance in the G protein and antigenic distance [42]. Therefore, it is reasonable that the best vaccine strain should be the one most closely related to the street strains circulating within the target area. Sequence analysis showed that compared to aG, PM and PV vaccine strains, which were widely used in China for human vaccine production, the CTNCEC25 strain was more closely related to RABV strains circulated in China while the other three vaccine strains were predominantly clustered with RABV strains derived from other countries. In addition, the ectodomain amino acid homology of the G proteins of CTNCEC25 with other RABV strains ranged from 90.0% to 99.1% (Table 5), which significantly ranked above the threshold 74% for the presence of cross-protection. Taken together, the above results indicated that CTNCEC25 was an ideal candidate for human vaccine production in China.

The human rabies vaccines can be produced either from animal tissues or cultured cells, such as CECs, BHK or Vero cells [43]. The development of modern industrial cell cultivation and fermentation techniques have greatly promoted the capacity of producing vaccines with high quantity and quality. Given the consideration of purity and concentration of vaccines, vaccines using cultured cells have quickly outdated the use of tissue-derived rabies vaccines. However, although cell culture vaccines are highly efficacious and immunogenic, these cell lines may have differences in genotypes or phenotypes from the original cell line and thus may contain oncogenic properties [44, 45]. Therefore, great caution should be taken in using such cell lines for vaccine manufacturing. Specific guidelines for producing human vaccines using the continuous cell lines were enacted in China and no more than 100 pg of host cellular DNA per dose was allowed for authorized vaccine production using Vero cell line according to the standard of the Pharmacopoeia of the People's Republic of China (2010), Volume III. On the other hand, CECs, which have limited life span than continuous cell lines, maintain the normal cellular karyotype and thus guarantee no contamination of foreign and oncogenic particles and are expected to be a promising substitute substrate for production of safe human vaccine [43]. The FluryLEP strain has already been adapted to CECs to produce purified chicken embryo cells vaccines, and has been recommended by WHO and widely used in many countries due to its high safety and efficacy, low cost and relative simple manufacturing techniques [46–49]. Current vaccine production in China was almost exclusively based on Vero cells, making vaccine strains adapted to CECs urgently needed.

## Conclusion

In this study, an CECs-adapted RABV strain CTNCEC25 was sequenced and characterized. Phylogenetic analysis identified that CTNCEC25 was more closely related to RABV street strains circulating in China than other RABV vaccine strains currently used in China. Sequence comparison showed that the G protein ectodomain amino acid sequence identity between CTNCEC25 and other RABV strains was at least 90% identical. Furthermore, CTNCEC25 produced high virus titers in primary culture cells while it lacked the pathogenicity for adult mice. Collectively, these results demonstrated that the CTNCEC25 strain is an ideal vaccine strain candidate for producing human vaccine with high quality and safety in China.

## Materials and methods

### Viruses and cells

The CTN-1 strain (after 5 passages in Vero cell lines) was obtained from National Institute for the Control of Pharmaceutical and Biological Products (NICPBP). The CTNCEC25 strain was prepared by adapting the CTN-1 strain to CECs through serial passage in CECs [32], and passage 36 of the CTNCEC25 strain in CECs was used in this study unless otherwise specified. BSR cells (cloned from BHK-21), CECs and Vero cells were maintained in M199 (Invitrogen) supplemented with 10% fetal bovine serum (FBS). Mouse neuroblastoma NA cells were grown in Eagle’s minimal essential medium supplemented with 10% FBS. All cells were incubated at 37°C in 5% CO_2_.

### Primer design

According to the conserved regions of the genome sequence of the CTN-1 strain published in GenBank (GenBank accession no. FJ959397), 13 pairs of primers were designed to amplify the regions of CTNCEC25 strain using DNASTAR 7.0 software (DNASTAR Inc., Madison, WI, USA) and synthesized and purified using PAGE purification by Invitrogen (Table 1).

### RNA extraction, reverse transcription-PCR and direct sequencing

Total RNA was extracted from supernatants of CTNCEC25 infected CECs using the QIAampViral RNA Mini Kit (Qiagen) according to manufacturer’s instructions. The first strand of cDNA was synthesized using the First Strand cDNA Synthesis Kit (TOYOBO) and the cDNA was amplified using the Platinum Taq DNA HIF I Polymerase (Invitrogen), according to the manufacturer’s protocols. Then, the full-length cDNA was subjected to nucleotide sequencing straightly by BGI-Beijing (Beijing, China) and Invitrogen Life Technologies Corporation (Shanghai, China) using the Chain Termination Method. The nucleotide sequence of the full-length genome of CTNCEC25 was submitted to GenBank under accession no. KJ466147.

### Sequence alignment and phylogenetic analysis

The nucleotide sequences obtained were edited manually using the DNASTAR 7.0. Multiple sequence alignments were constructed using Clustal X [50] with default settings. Phylogenetic trees were constructed on the basis of multiple alignments using the neighbor-joining algorithm in the software package MEGA 4. The reliability of the phylogeny groupings was evaluated using bootstrapping with 1000 replicates. The RABV strains with complete genome or the G gene sequence available in the GenBank used in this study were listed in Table 2.

### Virus titration

The virus titer was determined using a modified rapid fluorescence focus inhibition test as previously described [51] and expressed in FFUs/ml. Briefly, a monolayer of BSR cells in 96-well plates was incubated with serial three-fold virus dilutions. At 24 h p.i., the cells were fixed with 80% ice-cold acetone and stained with a FITC-labeled monoclonal antibody against nucleoprotein (Millipore) for 1 h at 37°C. The plates were examined by fluorescence microscopy, and the number of fluorescent foci presented in the wells was recorded. Endpoints were defined as the highest dilutions with fluorescent foci less than 30, and virus titers were calculated by the following formula: virus titer (FFU/ml) = (the mean foci number in the endpoint wells × 5 + the mean foci number in the wells with lower dilutions next to the endpoint well) ÷ 2 × the dilution factor of the lower dilutions × 20.

### Virus growth analysis in cultured cells

NA, Vero and CEC cells (1 × 10^6^) were infected with the indicated viruses at an MOI of 3 FFUs/ml for single-step growth analysis as previously described [39]. Briefly, after 1 h of incubation at 37°C, the inoculum was removed and cells were washed three times with Hanks’ balanced salt solution, then 3 ml of the corresponding fresh medium was added to the cells. Cells were incubated at 37°C in 5% CO_2_ and supernatants were harvested at the indicated time points. Titration of viral stocks was performed by an direct immunofluorescence assay as described above.

## Abbreviations

RABV: Rabies virus
CNS: Central nervous system
RNP: Ribonucleoprotein
CECs: Chick embryo cells
nt: Nucleotides
CDC: Coding sequence
FFU: Fluorescent focus unit
MOI: Multiplicity of infection
p.i.: Post infection
i.c.: Intracerebral
VNA: Virus-neutralizing antibody.
